# Fibroblast growth factor 21 as a circulating biomarker at various stages of colorectal carcinogenesis

**DOI:** 10.1038/s41416-018-0280-x

**Published:** 2018-11-14

**Authors:** Jing Qian, Kaja Tikk, Korbinian Weigl, Yesilda Balavarca, Hermann Brenner

**Affiliations:** 1 Division of Clinical Epidemiology and Aging Research, German Cancer Research Center (DKFZ), 69120 Heidelberg, Germany; 2 Medical Faculty Heidelberg, University of Heidelberg, 69120 Heidelberg, Germany; 3 German Cancer Consortium (DKTK), German Cancer Research Center (DKFZ), 69120 Heidelberg, Germany; 4 Division of Preventive Oncology, German Cancer Research Center (DKFZ) and National Center for Tumor Diseases (NCT), 69120 Heidelberg, Germany

**Keywords:** Cancer screening, Diagnostic markers

## Abstract

**Background:**

Despite evidence that inflammation and metabolism play a crucial role in colorectal carcinogenesis, there have been few studies on the association of inflammatory and metabolic protein biomarkers in various stages of colorectal carcinogenesis.

**Methods:**

Ninety-two inflammatory and metabolic biomarkers were measured in plasma samples of participants of screening colonoscopy. Markers identified to be significantly associated with the presence of advanced colorectal neoplasia (ACN) in a discovery set (*n* = 204) were validated in an independent replication set (*n* = 422). Adjusted associations with the presence of non-advanced adenomas (NAA), advanced precancerous lesions (APL) and colorectal cancer (CRC) were quantified by multiple logistic regression.

**Results:**

Out of the 92 inflammatory proteins, 72 markers were evaluable and 8 showed statistically significant associations with the odds of ACN after full adjustment for potential risk factors for CRC in the discovery set. One of these, fibroblast growth factor 21 (FGF-21), could be validated in the replication set. The multivariable-adjusted odds ratio (OR) reached 2.65 (95% CI, 1.50–4.81) for individuals with FGF-21 levels within the highest tertile, compared to those within the lowest tertile (*P*_trend_ across tertiles = 0.001). Separate models revealed fully adjusted ORs for NAA, APL and CRC of 2.99 (95% CI, 1.45–6.58, *P*_trend_ = 0.005), 2.24 (95% CI, 1.18–4.44, *P*_trend_ = 0.021) and 3.92 (95% CI, 1.51–12.18, *P*_trend_ = 0.003), respectively.

**Conclusions:**

Circulating FGF-21 level is associated with increased risk of early and late stages of colorectal carcinogenesis, supporting a role of inflammation and metabolism at all stages of colorectal carcinogenesis, and suggesting potential use of this biomarker for risk stratification in CRC screening.

## Introduction

Accumulating evidence suggests that inflammation plays a crucial role in colorectal carcinogenesis and progression.^1^ The susceptibility to colorectal cancer (CRC) is strongly increased in patients with ulcerative colitis and Crohn’s disease.^2^ A reduction in risk of colorectal adenomas and cancer was suggested among users of non-steroidal anti-inflammatory drugs (NSAIDs) in both observational studies^3,4^ and randomised controlled trials.^5–8^ Metabolic alteration is one newly discovered hallmark of cancer^9^ and metabolic reprogramming is suggested as one of the key initiating factors in colorectal tumourigenesis.^10^ Metabolic diseases, such as obesity, have been demonstrated to confer increased risk for CRC and regulate malignant transformation via inflammatory pathways.^11,12^ The putative relationship between metabolism, inflammation and colorectal carcinogenesis is shown in Figure S1.

Given the critical roles of inflammation and metabolism in sequential progression from normal mucosa to adenomatous polyps to CRC, identification of inflammatory and metabolic markers associated with risk of developing advanced colorectal neoplasia, including CRC and its precursors (advanced precancerous lesions), is of particular interest as it might expand insights regarding the underlying mechanisms linking inflammation and metabolism with colorectal carcinogenesis. More importantly, identification of inflammatory and metabolic markers associated with risk of advanced colorectal neoplasia could aid in defining individuals at high risk for CRC or its precursors, those who would be more likely to benefit from screening, such as screening colonoscopy.

Several circulating inflammatory and metabolic biomarkers or biomarker combinations have been identified as risk markers for CRC.^13–20^ Only a few markers, however, were found to be related to risk of colorectal adenoma.^21,22^ Taking advantage of ongoing screening studies in the general population, our current study aimed to explore the association of a wide range of circulating inflammatory and metabolic proteins with the risk of advanced colorectal neoplasia, as well as separate stages of colorectal carcinogenesis (including non-advanced adenomas, advanced precancerous lesions and CRC), and to validate those associations with an independent replication set.

## Materials and methods

### Study design and population

We employed a two-stage design with marker selection in a discovery set and validation in an independent replication set drawn from another study. Participants in the discovery set were drawn from the ASTER study, a multicentre, prospective randomised placebo-controlled trial (EudraCT No.2011-005603-32). This trial recruited men and women aged 40–80 years with a planned screening or diagnostic colonoscopy from gastrointestinal practices in Southern Germany between 2014 and 2016. The primary objective of the trial was to determine whether administration of a single dose of 300 mg of acetylsalicylic acid (ASS) prior to conduction of faecal immunochemical test (FIT) would improve sensitivity of FIT for detecting advanced colorectal neoplasia. Patients were recruited and blood samples were taken in gastrointestinal practices prior to administration of the study medication (300 mg ASS or placebo) and colonoscopy. In addition, patients were asked to fill out a standardised questionnaire on demographics, personal lifestyles and medical history. Medical data, particularly previous colonoscopy findings, were additionally obtained from colonoscopy and histology records.

**Fig. 1:**
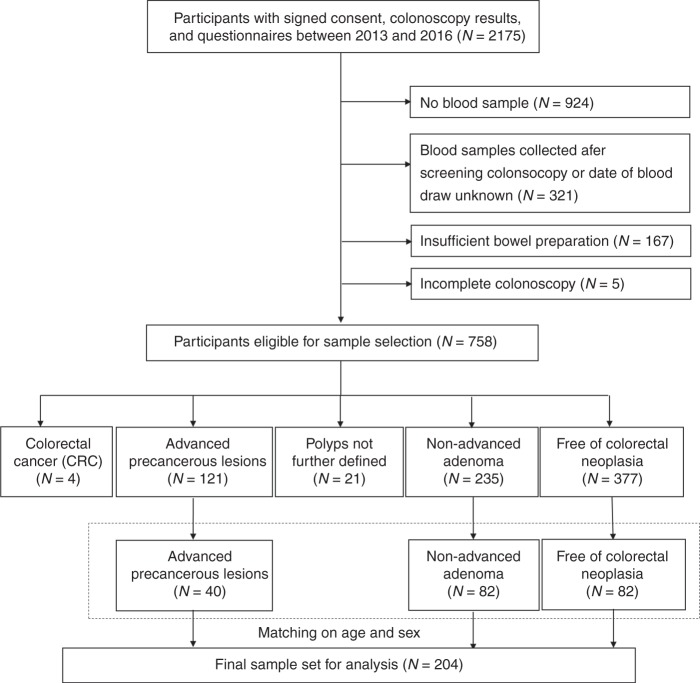
Flowchart of participant selection from the ASTER study

Of the 2175 participants enrolled in the study between 2013 and 2016, participants with missing blood samples, blood samples collected after screening colonoscopy or with unknown date of blood draw, with insufficient bowel preparation, or incomplete colonoscopy were excluded (Fig. 1). Out of the eligible 121 participants with advanced precancerous lesions (including advanced adenomas or sessile serrated lesions ≥1 cm in size) that were detected via colonoscopy, 41 cases were selected by simple random sampling to ensure representativeness of the population with advanced precancerous lesions in the original study. Advanced adenomas were defined as high grade or severe dysplasia, adenomas with villous or tubulovillous characteristics or adenomas ≥1 cm. Furthermore, 82 eligible participants with non-advanced adenoma and 82 participants free of neoplasia were selected, employing frequency matching by sex and age to the group of advanced colorectal neoplasia participants. One advanced precancerous lesion case was excluded for the final analysis due to failure of sample quality control.

Independent replication was performed in the BLITZ study, an ongoing study among participants undergoing screening colonoscopy. It is conducted in cooperation with 20 gastroenterology practices in Southern Germany since November 2005. Details on this study have been reported elsewhere.^23–25^ In brief, participants were asked to provide blood samples and complete a standardised questionnaire on demographics, personal lifestyles and medical history prior to screening colonoscopy. Medical data, colonoscopy findings in particular, were additionally obtained from colonoscopy and histology reports.

**Fig. 2:**
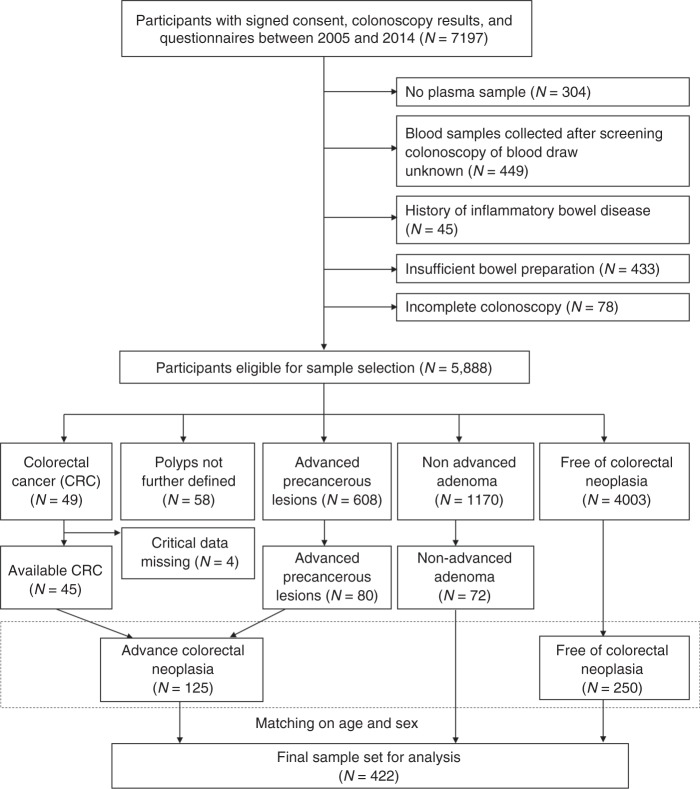
Flowchart of participant selection from the BliTz study

Figure 2 shows the selection of BLITZ participants for this analysis. The same exclusion criteria outlined above were employed as in ASTER. We additionally excluded participants with a history of inflammatory bowel diseases who had not been recruited in ASTER in the first place. Among 5888 eligible participants recruited between 2005 and 2014, all 45 cases with CRC were included in the analysis, and 80 cases with advanced precancerous lesion were randomly selected from 608 eligible patients with advanced precancerous lesion. Two hundred and fifty control participants free of neoplasia were frequency matched by sex and age to the 125 participants with advanced colorectal neoplasia. In addition, 72 participants were randomly selected from eligible subjects with non-advanced adenoma.

To be noted, discovery and replication set-up in our study refer to the strategy for data analysis. Since our focus was on discovery of reasonably strong markers that could only be detected in a small sample and reliably validated in a large sample, we chose the smaller data set for discovery and the larger sample for validation. This approach also allowed additional subgroup analyses of interest, with reasonable sample size in the latter.

Both studies were approved by the Ethics Committee of the University of Heidelberg (Heidelberg, Germany) and by the Ethics Committee of each participating centre. Written informed consent was obtained from all participants.

### Data collection

Self-administrated questionnaires used in both studies included detailed information on age, sex, nationality, residence, education, body size, diet, physical activity, smoking status, alcohol consumption, medical history, family history of cancer in first-degree relatives, history of endoscopy and regular use of aspirin and NSAIDs, as described before.^23,25^ After colonoscopy was performed, colonoscopy and histology reports were collected, and data were extracted in a standardised manner by two trained independent research assistants.

### Specimen collection and storage

Plasma samples were taken in ethylenediaminetetraacetic acid-containing tubes and immediately centrifuged at 2123 × *g* for 10 min at 4 °C. Then the supernatant was transferred into new tubes and transported to the biobank at German Cancer Research Center in a cold chain. The median time between blood withdrawal and arrival of samples at the biobank was 1 day. Once arriving at the biobank, plasma samples were again centrifuged at 2123 × *g* for 10 min at 4 °C, divided into several aliquots and stored at −80 °C until measurement. The laboratory personnel were blinded to information regarding the study population.

### Laboratory measurement and data preprocessing

Protein in plasma was profiled with the Proseek Multiplex Inflammatory I Kit (Olink Bioscience) and quantified by the Fluidigm BioMark HD real-time PCR platform, as described previously.^26^ In order to ensure consistency of laboratory measurements in the discovery and validation set, the same analytic platform was used for both data sets. The panel permits analysis of 92 protein biomarkers related to inflammatory and metabolic processes across 96 samples simultaneously (the full list of proteins is shown in Table S1). Details on the reliability and stability of the technology and the potential factors influencing the levels of biomarkers are provided in Supplementary Method and Table S1. Normalisation of raw data was conducted as described previously.^26,27^ To correct for batch effect in Proximity Extension Assays (PEA) measurement, the ComBat approach^28^ was adopted in Bioconductor under R environment (version 3.3.0) (R Core Team). The data for analyses was presented as Normalised Protein eXpression (NPX). LOD (limit of detection) was defined as mean NPX value of the three negative controls plus 3 × standard derivations (SDs). Markers with >65% of values below the LOD were excluded from the analyses. Of the 92 proteins measured by PEA in the entire study population, 72 proteins with ≤65% values below LOD were regarded as evaluable markers. Data with NPX value below the LOD were replaced with LOD when <15% of the observations were not detected (3% after exclusion of 20 proteins in the current study) according to recommendation by Guidance for Data Quality Assessment (United States Environmental Protection Agency 2000).

### Statistical analysis

Circulating biomarker levels (NPX levels) were compared between carriers of advanced colorectal neoplasia and controls, as well as carriers of non-advanced adenoma and controls using Wilcoxon rank-sum test. Then NPXs were categorised into tertiles based on the distribution among control subjects in the whole population. To determine the association of individual markers with advanced colorectal neoplasia, age- and sex-adjusted and multivariable logistic regression models were used to estimate odds ratios (OR) and 95% confidence intervals (CIs). Apart from the matching factors, we also adjusted for other potential confounding factors, including body mass index (BMI, continuous variable), red meat intake, smoking status, alcohol intake, history of CRC in first-degree relatives and history of colonoscopy. Linear trends were tested using a continuous variable with values of 1, 2, 3 for the respective tertiles of the proteins in the logistic regression model. To examine potential heterogeneity with regards to the association between biomarkers and CRC, advanced precancerous lesions and non-advanced adenoma, separate multivariable-adjusted logistic models were run in the replication set.

Stratified analyses by age were conducted, and interactions between age and the individual potential markers associated with the presence of advanced neoplasia were tested by including cross-product terms between age (continuous) and continuous values of individual markers in multiple logistic regression models and evaluating them with a likelihood ratio test. As regular use of NSAIDs is one of the exclusion criteria for the ASTER study, it was not included as covariate in multiple logistic regression models in both sets for comparability. However, given that regular use of NSAIDs is a potent protective factor for advanced colorectal neoplasia, this factor was additionally adjusted for in multiple logistic regression in sensitivity analyses using the replication set.

To investigate the diagnostic performance of the identified and validated protein markers, the area under the receiver operating characteristic curve (AUC) and sensitivity at 80% and 90% specificity were estimated. 95% CIs of AUC and sensitivity were derived based on 1000 bootstrap samples.

All statistical tests were two-sided and *P* < 0.05 was considered statistically significant. In order not to miss potentially relevant markers, no correction for multiple testing was employed when identifying and selecting markers potentially associated with the risk of advanced colorectal neoplasia in the discovery set. However, the Benjamini–Hochberg correction for multiple testing was used when validating the selected potential markers in the replication set, to exclude potentially false positive markers identified in the discovery set. All analyses were performed with R language and environment (version 3.3.0, R Core Team) for statistical computing.

## Results

### Baseline characteristics of study population

Baseline characteristics of the study population are displayed in Table 1. The distribution of characteristics was largely similar between the discovery set and the replication set, except that a much larger proportion of participants in the discovery set, who were recruited in more recent years, reported a previous colonoscopy than in the replication set. Compared with control subjects free of neoplasia, advanced colorectal neoplasia cases were more likely to smoke in both sets. In addition, advanced colorectal neoplasia patients in the replication set tended to drink more alcohol than controls.

**Table 1:** Baseline characteristics of study population

Variables	Discovery set									Replication set							
	Advanced colorectal neoplasia (*N* = 40)			Non-advanced adenoma (*N* = 82)			Control (*N* = 82)			Advanced colorectal neoplasia (*N* = 125)			Non-advanced adenoma (*N* = 72)			Control (*N* = 250)	
	*n*	%	*P* ^a^	*n*	%	*P* ^b^	*n*	%	*n*		%	*P* ^a^	*n*	%	*P* ^b^	*n*	%
Age (years)																	
<60	11	27.5	1.000	23	28.0	0.980	24	29.3	42		33.6	1.000	27	37.5	0.931	84	33.6
60–64	10	25.0		21	25.6		20	24.4	34		27.2		17	23.6		66	26.4
65–69	8	20.0		14	17.1		16	19.5	24		19.2		13	18.1		49	19.6
≥70	11	27.5		24	29.3		22	26.8	25		20.0		15	20.8		51	20.4
Median (IQR)	64 (59–71)			64 (58–70)			64 (58–70)		63 (58–69)				62 (58–70)			63 (58–70)	
Sex																	
Female	17	42.5	1.000	35	42.7	1.000	36	43.9	61		48.8	1.000	29	40.3	0.229	122	48.8
Male	23	57.5		47	57.3		46	56.1	64		51.2		43	59.7		128	51.2
Cigarette smoking^c^																	
Never	21	52.5	0.022	37	45.7	0.760	39	48.1	51		40.8	0.010	34	47.2	0.445	136	55.3
Former	10	25.0		35	43.2		36	44.4	54		43.2		32	44.4		90	36.6
Current	9	22.5		9	11.1		6	7.4	20		16.0		6	8.3		20	8.1
Alcohol intake^d^																	
Non-risky	30	78.9	0.510	52	66.7	0.490	58	72.5	76		60.8	0.047	53	73.6	0.767	178	71.2
Risky^e^	8	21.1		26	33.3		22	27.5	49		39.2		19	26.4		72	28.8
Red meat intake^f^																	
~1 time/day	38	95.0	1.000	77	93.9	1.000	77	93.9	119		95.2	0.150	65	92.9	0.810	223	90.7
>1 time/day	2	5.0		5	6.1		5	6.1	6		4.8		5	7.1		23	9.3
Regular use of NSAIDs																	
No	—	—	—	—	—	—	—	—	99		79.2	0.400	53	73.6	0.812	207	82.8
Yes	—	—		—	—		—	—	26		20.8		19	26.4		43	17.2
Family history of CRC (first-degree relative)^g^																	
No	30	75.0	0.820	63	76.8	1.000	64	78.0	100		82.0	0.070	62	86.1	0.091	219	89.0
Yes	10	25.0		19	23.2		18	22.0	22		18.0		10	13.9		27	11.0
History of colonoscopy																	
No	19	46.3	0.073	26	31.7	0.870	24	29.3	100		80.0	0.364	49	68.1	0.224	189	75.6
Yes	22	53.7		56	68.3		58	70.7	25		20.0		23	31.9		61	24.4
BMI (kg/m^2^)^h^, median (IQR)	26.3 (24.4–29.1)		0.496	26.3 (24.1–28.7)		0.410	25.4 (23.6–29.4)		26.6 (24.1–29.5)			0.064	26.2 (24.8–29.4)		0.064	25.9 (23.5–28.1)	

^a^The difference of variables between advanced colorectal neoplasia and control was compared and two-sided *P* value was estimated with Fisher exact test for categorical variables and Wilcoxon rank-sum test for continuous variables

^b^The difference of variables between non-advanced adenoma and control was compared and two-sided *P* value was estimated with Fisher exact test for categorical variables and Wilcoxon rank-sum test for continuous variables

^c^Data missing in 2 and 4 subjects, respectively, in the discovery and replication sets

^d^Data missing in 8 subjects in the discovery set

^e^Alcoholic consumption was defined as risky when a person drank >5 days per week or drank >7 (women) or >14 (men) standard alcoholic beverages per week. One standard alcoholic beverage equals a small portion of beer (0.25 l) or wine (0.1 l) or 4 cl schnapps

^f^Data missing in 6 subjects in the replication set

^g^Data missing in 7 subjects in the replication set

^h^Data missing in 2 and 7 subjects, respectively, in the discovery and replication sets

### Plasma biomarker levels and various stages of colorectal carcinogenesis

Twenty proteins with >65% of values below the LOD were excluded from the analyses, and a brief description of these markers on their types and functions are listed in Table S2. Most of the proteins at the very low levels were inflammation-related cytokines and only two were growth factors involved in regulation of neural development and survival. Fourteen proteins belonging to the interleukin family were found, making them the most common category.

In the discovery set, 8 markers out of the 72 evaluable proteins showed significant associations with odds of advanced colorectal neoplasia accounting for age and sex (Table 2): six markers (monocyte chemotactic protein 3 [MCP-3], CUB domain-containing protein 1 [CDCP1], latency-associated peptide [LAP], interleukin-6 [IL-6], chemokine C-C motif ligand 4 [CCL4], fibroblast growth factor 21 [FGF-21]) were positively associated with increased odds of advanced colorectal neoplasia, while two markers (tumour necrosis factor (TNF)-related apoptosis-inducing ligand [TRAIL], stem cell factor [SCF]) showed opposite associations. Associations did not materially change after adjustment for multiple covariates. Among the eight proteins, none showed significant association with likelihood of developing non-advanced adenomas.

**Table 2:** Markers associated with risk of advanced colorectal neoplasia in the discovery set

Markers	Tertile	Case no.	Control no.	Model 1^a^		Model 2^b^	
				OR (95% CI)	*P*_trend_^c^	OR (95% CI)	*P*_trend_^c^
MCP-3	1	5	20	Ref.	0.043	Ref.	0.048
	2	21	45	1.92 (0.66–6.42)		1.87 (0.65–6.21)	
	3	14	17	3.42 (1.06–12.58)		3.29 (1.03–11.95)	
CDCP1	1	9	33	Ref.	0.015	Ref.	0.014
	2	12	28	1.56 (0.57–4.42)		1.57 (0.58–4.38)	
	3	19	21	3.32 (1.27–9.22)		3.32 (1.29–9.02)	
LAP (TGFβ-1)	1	3	18	Ref.	0.017	Ref.	0.019
	2	13	31	2.58 (0.71–12.38)		2.52 (0.70–12.05)	
	3	24	33	4.59 (1.34–21.39)		4.36 (1.29–20.14)	
IL-6	1	12	40	Ref.	0.039	Ref.	0.036
	2	12	22	1.80 (0.69–4.75)		1.82 (0.70–4.77)	
	3	16	20	2.64 (1.05–6.81)		2.67 (1.07–6.83)	
TRAIL	1	20	25	Ref.	0.026	Ref.	0.024
	2	11	24	0.56 (0.21–1.42)		0.57 (0.22–1.43)	
	3	9	33	0.34 (0.13–0.87)		0.34 (0.13–0.86)	
CCL4	1	6	31	Ref.	0.015	Ref.	0.017
	2	17	28	3.35 (1.19–10.55)		3.14 (1.13–9.72)	
	3	17	23	4.03 (1.41–12.83)		3.82 (1.36–12.00)	
SCF	1	24	21	Ref.	0.001	Ref.	0.001
	2	10	32	0.26 (0.10–0.65)		0.27 (0.11–0.67)	
	3	6	29	0.18 (0.06–0.49)		0.18 (0.06–0.50)	
FGF-21	1	6	33	Ref.	0.025	Ref.	0.022
	2	18	25	4.17 (1.49–13.11)		3.96 (1.43–12.27)	
	3	16	24	3.54 (1.25–11.20)		3.67 (1.30–11.51)	

*no.*, number; *ref.*, reference; *OR*, odds ratio; *CI*, confidence interval

^a^Model 1 adjusted for age (continuous variable) and sex

^b^Model 2 adjusted for age (continuous variable), sex, BMI (continuous variable), red meat intake, smoking status, alcohol intake, CRC family history of first-degree relative and history of colonoscopy

^c^The test of linear trend was performed using a continuous variable with values of 1, 2, 3 for the tertiles in the logistic regression model

The NPX levels of each of the eight markers in the discovery and the replication sets are shown as a boxplot plot in Figure S2. All of the eight biomarkers showed significantly different plasma levels between advanced colorectal neoplasia cases and controls in the discovery set, while only FGF-21 showed similar trends in the replication set. The levels between non-advanced adenoma and controls were mostly similar with exception to FGF-21 in the replication set. The levels of FGF-21 increased with the malignancy of polyps (control→non-advanced adenoma→advanced colorectal neoplasia) (*P* = 0.034 and *P* < 0.001 in the discovery and replication set, respectively).

Among the eight markers associated with advanced colorectal neoplasia odds in the discovery set, only FGF-21 could be validated in the replication set with an age- and sex-adjusted OR of 2.65 for the highest versus lowest tertile (95% CI 1.50–4.84) and of 1.83 for the middle versus the lowest tertile (95% CI 1.00–3.41, *P*_trend_ = 0.001) (Table 3). The associations remained essentially unchanged by additional adjustment for multiple covariates. Statistical significance clearly persisted after correction for multiple testing (*P* = 0.008).

**Table 3:** Risk of advanced colorectal neoplasia according to plasma levels of eight markers in the replication set

Markers	Tertile	Case no.	Control no.	Model 1^a^			Model 2^b^		
				OR (95% CI)	*P*_trend_^c^	Adjusted *P*^d^	OR (95% CI)	*P*_trend_^c^	Adjusted *P*^d^
MCP-3	1	40	82	Ref.	0.831	0.950	Ref.	0.733	0.838
	2	46	84	1.12 (0.66–1.89)			1.00 (0.57–1.74)		
	3	39	84	0.94 (0.55–1.62)			0.91 (0.51–1.60)		
CDCP1	1	33	78	Ref.	0.25	0.950	Ref.	0.246	0.838
	2	40	82	1.16 (0.66–2.05)			1.15 (0.66–2.02)		
	3	52	90	1.40 (0.79–2.52)			1.37 (0.81–2.34)		
LAP-TGFβ-1	1	43	93	Ref.	0.975	0.975	Ref.	0.964	0.964
	2	46	79	1.26 (0.75–2.10)			1.26 (0.75–2.11)		
	3	36	78	0.99 (0.58–1.70)			1.00 (0.58–1.70)		
IL-6	1	37	71	Ref.	0.685	0.950	Ref.	0.653	0.838
	2	36	88	0.78 (0.45–1.37)			0.79 (0.45–1.37)		
	3	52	91	1.09 (0.64–1.86)			1.10 (0.65–1.86)		
TRAIL	1	44	86	Ref.	0.600	0.950	Ref.	0.587	0.838
	2	46	84	1.07 (0.64–1.80)			1.07 (0.64–1.79)		
	3	35	80	0.86 (0.50–1.47)			0.86 (0.50–1.46)		
CCL4	1	42	79	Ref.	0.740	0.950	Ref.	0.726	0.838
	2	32	82	0.73 (0.41–1.27)			0.73 (0.42–1.27)		
	3	51	89	1.07 (0.64–1.79)			1.08 (0.65–1.80)		
SCF	1	46	90	Ref.	0.525	0.950	Ref.	0.538	0.838
	2	42	74	1.11 (0.66–1.87)			1.11 (0.66–1.87)		
	3	37	86	0.84 (0.49–1.41)			0.84 (0.50–1.42)		
FGF-21	1	21	78	Ref.	0.001	0.008	Ref.	0.001	0.008
	2	42	85	1.83 (1.00–3.41)			1.84 (1.01–3.41)		
	3	62	87	2.65 (1.50–4.84)			2.65 (1.50–4.81)		

*no.*, number; *ref.*, reference; *OR*, odds ratio; *CI*, confidence interval

^a^Model 1 adjusted for age (continuous variable) and sex

^b^Model 2 adjusted for age (continuous variable), sex, BMI (continuous variable), red meat intake, smoking status, alcohol intake, CRC family history of first-degree relative and history of colonoscopy

^c^The test of linear trend was performed using a continuous variable with values of 1, 2, 3 for the tertiles in the logistic regression model

^d^False discovery rate was adjusted using the Benjamini–Hochberg correction

Table 4 shows separate risk estimations for the association of FGF-21 with CRC, advanced precancerous lesion and non-advanced adenoma in the replication set. Clear dose–response relationships were seen with each of the different outcomes, with ORs (95% CI) for the highest versus lowest tertile of 3.92 (1.51–12.18), 2.24 (1.18–4.44) and 2.99 (1.45–6.58), respectively (multiple testing adjusted *P* value = 0.008, 0.021 and 0.008, respectively).

**Table 4:** Risk of colorectal cancer, advanced precancerous lesions and non-advanced adenomas according to plasma levels of FGF-21 in the replication set

	Tertile 1	Tertile 2	Tertile 3	*P*_trend_^a^	Adjusted *P*^b^
Colorectal cancer					
No. (case/control)	5/78	7/85	23/87		
OR (95% CI)^c^	Ref.	1.34 (0.41–4.71)	3.92 (1.51–12.18)	0.003	0.008
Advanced precancerous lesions					
No. (case/control)	16/78	35/85	39/87		
OR (95% CI)^c^	Ref.	1.98 (1.03–3.96)	2.24 (1.18–4.44)	0.021	0.021
Non-advanced adenomas					
No. (case/control)	11/78	26/85	35/87		
OR (95%CI)^c^	Ref.	2.29 (1.08–5.15)	2.99 (1.45–6.58)	0.005	0.008

*no.*, number; *ref.*, reference; *OR*, odds ratio; *CI*, confidence interval

^a^The test of linear trend was performed using a continuous variable with values of 1, 2, 3 for the tertiles in the logistic regression model

^b^False discovery rate was adjusted using the Benjamini–Hochberg correction

^c^The model was adjusted for age (continuous variable), sex, BMI (continuous variable), red meat intake, smoking status, alcohol intake, CRC family history of first-degree relative and history of colonoscopy

As FGF-21 has been recently strongly linked to ageing, age-specific analyses were also conducted in the replication set (Table S3). Associations of FGF-21 level with odds of advanced colorectal neoplasia were observed for both younger and older participants with borderline significance, while FGF-21 level was associated with odds of non-advanced adenoma for younger individuals. However, no interaction was found between age and FGF-21 in relation to the odds of both advanced colorectal neoplasia and non-advanced adenoma (*P*_interaction_ = 0.630 and 0.837, respectively). In sensitivity analyses, additional adjustment for regular use of NSAID in the replication set did not materially change the estimates for either advanced colorectal neoplasia or non-advanced adenoma (Table S4).

### Diagnostic performance of FGF-21 to detect CRC and its precursors

The AUC of the plasma FGF-21 to detect advanced colorectal neoplasia in the replication set was 0.61 (95% CI 0.55–0.67) with 24.8% sensitivity at 80% specificity and 18.4% sensitivity at 90% specificity (Table S5). The diagnostic performance of the biomarker was much better for CRC compared with advanced precancerous lesion as well as non-advanced adenoma, yielding an AUC (95% CI) of 0.71 (0.61–0.81) with 45.7% sensitivity at 80% specificity and 37.1% sensitivity at 90% specificity. The AUC for non-advanced adenoma was similar to that of advanced colorectal neoplasia, though FGF-21 showed slightly higher sensitivity to detect advanced colorectal neoplasia than non-advanced adenoma.

## Discussion

To explore the relationship of inflammation and metabolism with various stages of colorectal carcinogenesis, we measured multiple circulating proteins, representing different inflammatory and metabolic processes, in a screening study. We found a strong association between fibroblast growth factor (FGF-21) and odds of advanced colorectal neoplasia, independent of several established risk factors for ACN. These results were validated in an independent prospective study, in which strong associations of circulating FGF-21 and risk of CRC, as well as early and advanced precursors of the disease, were confirmed.

FGF-21, as a member of an atypical subfamily of FGFs, acts as a hormone to modulate metabolism of glucose and lipids and metabolic adaptation when released into systemic circulation from the liver, muscles and adipose tissue.^29^ Also, FGF-21 is involved in inflammation and immune homeostasis by prevention of age-related thymic degeneration^30^ and modulation of inflammatory mediators in the progression of colitis.^31^ Given the close link between CRC and inflammation and metabolism, it is rational to infer that FGF-21 might be also related to colorectal carcinogenesis. Several studies provided experimental evidence that circulating FGF-21 is implicated in cancer pathogenesis. Recent in vivo studies revealed that hepatic FGF-21 expression is activated and serum FGF-21 level is elevated in response to hepatocarcinogenesis.^32^ In addition, FGF21 could be induced by fasting and upregulate peroxisome proliferator-activated receptor gamma coactivator 1-α (PGC1α) in the liver.^29^ PGC1α is a key coordinator of metabolic pathways that convert glucose to fatty acids. Consequently, strengthened lipogenesis accelerates tumour growth and disrupts the balance between apoptosis and cell proliferation, thus promoting the development of malignancies.^33^ Furthermore, cancer cells have a high metabolic rate and thus can easily experience nutrient starvation.^9,34^ FGF-21 could be easily induced by nutrient starvation during cancer progression, then released into peripheral circulation.^29^ This may also partially explain the reason for increased risk of CRC among individuals with elevated FGF-21 levels. Our findings provide preliminary epidemiological evidence for the role of inflammation and metabolism in the development of CRC. However, the mechanism underlying the relationship between FGF-21 elevation and advanced colorectal neoplasia risk and the exact contribution of inflammation and metabolism in colorectal malignant transformation remain to be further clarified.

Few population-based studies have been conducted to investigate the role of FGF-21 in cancer. Akyol et al. observed marked reduction of FGF-21 levels in early breast cancer patients after adjuvant endocrine therapy with tamoxifen and aromatase.^35^ The team also reported that FGF-21 levels were elevated in patients with breast cancer since the early stages of the disease and showed a sensitivity of 95% and a specificity of 50% for the discrimination of breast cancer patients from controls.^36^ A recent proteomic profiling of pre-diagnostic plasma of 138 patients, using the same method as this study, also found FGF-21 to be associated with increased CRC risk,^37^ even though the estimated OR was lower (1.71) than in our study. To our knowledge, our study provides the first demonstration of a strong positive association of plasma FGF-21 levels with risk of advanced colorectal neoplasia, though the results need to be further replicated.

Our study is also the first to assess and disclose an association of FGF-21 with various stages of colorectal carcinogenesis. Though it is difficult to explain the reason underlying the stronger association of FGF-21 with risk of non-advanced adenoma than with risk of advanced precancerous lesion, the increment of FGF-21 with progression of colorectal neoplasms from control, non-advanced adenoma to advanced colorectal neoplasia and consistent findings of possible associations with non-advanced adenoma, advanced precancerous lesion and CRC collectively suggest that FGF-21 may be involved in inflammatory and metabolic process at both early and late stages of colorectal tumourigenesis. However, further research is required to elucidate potential underlying mechanisms.

A body of evidence demonstrated a crucial role for FGF-21 in adaptation of glucose and lipid metabolism and an involvement in lifestyle-related metabolic diseases.^29,38^ Given the cross-sectional nature of our data, it is impossible to distinguish potential effects of FGF-21 on the risk of colorectal neoplasia from potential modification of FGF-21 during the course of colorectal tumourigenesis, possibly even as a result of tumour development or metabolism-related risk factors, such as alcohol consumption, red meat intake or obesity. However, adjustment for these risk factors hardly changed the association between FGF-21 and occurrence of colorectal neoplasia, suggesting an independent relationship with the disease.

Apart from pointing to potential mechanism in colorectal carcinogenesis, our results may also have relevance for risk stratification in CRC screening. For example, the association between FGF-21 and colorectal neoplasia identified in our study is even substantially stronger than the well-established association between family history of CRC and colorectal neoplasia. Family history of CRC is so far the most frequently, or even the only, factor used for risk discrimination in CRC screening for defining starting age at screening, and such risk stratification might be greatly enhanced in the future by considering additional, potentially even stronger risk predictors, such as more comprehensive scores based on easy-to-collect risk factors,^39^ genetic risk scores^40^ or protein biomarkers like FGF-21. Furthermore, although FGF-21 is not strong enough as a screening marker alone and might be influenced by metabolic and inflammatory conditions, its potential to be a promising biomarker as part of a multi-biomarker blood-based panel for CRC screening should be further explored.

Several biomarkers previously reported to be associated with cancers failed to show associations with advanced colorectal neoplasia risk in our study. The most commonly studied IL-6, for instance, was not linked to advanced colorectal neoplasia risk after external validation, even though it showed up in the discovery set of our study. Although associations between CRC or adenoma risk and circulating IL-6 levels^41,42^ were demonstrated in few studies with limited sample size, some large cohort studies did not find an increased risk of CRC and adenoma in individuals with elevated IL-6 levels,^14,18,19,43^ which is in line with our findings. IL-8 is a multifunctional pro-inflammatory cytokine and promotes migration, invasion, proliferation and angiogenesis in cancers.^44^ Although a few small case–control studies with hospital-detected CRC patients suggested a link to CRC,^45,46^ no previous cohort study confirmed its association with CRC risk. For many other proteins measured in our study that have been suggested to be involved in CRC development in preclinical laboratory studies, study settings could be one of the plausible interpretation of the difference. Some preliminary in vitro and in vivo studies aiming to discover novel inflammatory markers involved in colorectal carcinogenesis commonly evaluate the clinical significance of biomarkers in clinical settings, in which clinically detected cases were individuals recruited from hospitals. As clinically diagnosed patients often differ dramatically from screening-detected cases in terms of many factors, such as preceding diagnostic procedures or even psychosocial consequences, it is not surprising that we cannot replicate the markers identified in the clinical settings. Besides, the majority of proteins at the very low levels in our study are cytokines. As no study has examined the pre-analytical factors influencing the stability of these specific cytokines, it is difficult to exclude the possibility of protein degradation during the sample handling and processing despite our Standard Operating Procedure. Therefore, future investigations on the potential importance of sample handling and processing on the levels of cytokines are desirable. To be noted, some inflammatory and metabolic molecules commonly investigated in several related epidemiological studies, such as TNF- receptor 2, C-reactive protein, TNF-α, insulin-like growth factor and leptin, were not included in our panel and direct comparison of the association between advanced colorectal neoplasia risk and these proteins with FGF-21 should be considered in further studies.

A major strength of our study is that it is based on two large study populations from true screening settings, with a rigorous two-step approach—a discovery step and external validation in an independent replication set. However, despite the overall large size of the study populations, the numbers of participants with advanced colorectal neoplasia, in particular those with CRC, were still quite limited—a feature commonly encountered in screening settings. In spite of the sample size limitation, consistent strong associations of FGF-21 with the various end points could still be confirmed even after correction for multiple testing in the replication set. Nevertheless, replication in larger studies, ideally with repeated longitudinal measurements of FGF-21, are needed to more fully understand a potential role of FGF-21 in colorectal carcinogenesis or to determine its role in risk stratification of CRC screening. Also, fasting status, which is unavailable in our data sets, might influence the levels of metabolic proteins and should also be considered in further studies.

In conclusion, circulating FGF-21 was associated with increased risk of early and late stages of colorectal carcinogenesis. Our data suggest that FGF-21 might be a promising marker for risk stratification to facilitate identification of high-risk candidates in whom initiation of CRC screening at younger ages or more intensive CRC screening might be warranted. Further population-based studies are needed to replicate these findings. Functional investigations and repeated, longitudinal measures of this inflammatory and metabolic protein might help to elucidate its exact contribution to cancer susceptibility and may help to identify potential novel, preventive, diagnostic and therapeutic targets.

## Electronic supplementary material

supplementary information_final

## Data Availability

The data sets used and analysed during the current study are available from the corresponding author.
