# Supplementary material for: Fibroblast growth factor 21 as a circulating biomarker at various stages of colorectal carcinogenesis
Source: Br J Cancer. 2018 Nov 14;119(11):1374–82. doi: 10.1038/s41416-018-0280-x (PMC6265243; doi:10.1038/s41416-018-0280-x)

**Supplementary information**

*Materials and methods*

**Laboratory measurement and data preprocessing**

The reliability (repeatability) of the Proximity Extension Assay was assessed by using intra-assay variation and inter-assay variation. Intra-assay variation was calculated as the mean coefficient of variation (CV) for 7 individual samples, within each of 10 separate runs. Inter-assay variation was calculated as the mean CV, for the same 7 individual samples, between 6 separate runs. Variation calculations were assessed on linearized values for all 92 analytes (Table S1). Across all 92 assays, the mean intra-assay and inter-assay variations were 7% and 18%, respectively. The stability over time was examined by correlation test in the same dataset from two time points. Eighty-nine of 92 proteins showed a significant correlation between two time points and the 3 proteins with significant correlation were below LOD for most samples.

The potential endogenous interference from heterophilic antibodies, e.g. human anti-mouse antibody (HAMA) and rheumatoid factor (RF), together with specific interfering substances (hemolysate, lipids and bilirubin) are potential factors that might influence the biomarker levels. Interference from HAMA and RF were evaluated by a special “mismatch” system and the only way to generate a signal in this system is by antibody probe pairs being brought into proximity, by cross-binding substances other than antigens. The test using the system showed that no interference interpreted as signal above LOD due to HAMA or RF disturbances could be detected for any of the samples, indicating a sufficient blocking ability in all assays in the Olink Inﬂammation I. The potential impact of certain known interfering serum and plasma components was evaluated by using serial dilutions of hemolysate, lipids and bilirubin, respectively. Table S1 lists the highest concentration of each interfering substance without impact on assay performance. In 10 out of 92 assays altered expression was observed by the addition of hemolysate. But hemolytic samples were excluded in our study. A concentration of 15 g/L of hemolysate represents 10% hemolysis of a sample. Also, in 10 assays interference was observed by addition of lipids ≥5 mg/mL, which would correspond to very high serum triglyceride levels. Addition of bilirubin altered 37 out of 92 assays at ≥79 µg/mL, which is more than 4 times the normal total bilirubin levels. To conclude, the common factors play a small role in determination of biomarker levels using PEA (The assay we used).

Table S1. Repeatability and Endogenous interference of the inflammation kit

| Protein | Repeatability | | Endogenous interference^a^ | | |
| --- | --- | --- | --- | --- | --- |
|  | Intra-assay variation (%) | Inter-assay variation (%) | Hemolysate (g/L) | Lipids (mg/mL) | Bilirubin (µg/mL) |
| Adenosine Deaminase (ADA) | 5 | 29 | 15 | 20 | 630 |
| Artemin (ARTN) | 7 | 18 | 15 | 20 | 630 |
| Axin-1 (AXIN1) | 6 | 19 | 15 | 20 | 630 |
| Beta-nerve growth factor (Beta-NGF) | 6 | 14 | 15 | 20 | 630 |
| Brain-derived neurotrophic factor (BDNF) | 6 | 10 | 15 | 20 | 630 |
| Caspase 8 (CASP-8 ) | 7 | 22 | 15 | 20 | 630 |
| C-C motif chemokine 4 (CCL4 ) | 6 | 17 | 7.5 | 20 | 315 |
| C-C motif chemokine 19 (CCL19) | 8 | 15 | 7.5 | 20 | 315 |
| C-C motif chemokine 20 (CCL20) | 7 | 13 | 15 | 20 | 158 |
| C-C motif chemokine 23 (CCL23) | 6 | 13 | 15 | 20 | 315 |
| C-C motif chemokine 25 (CCL25) | 6 | 18 | 15 | 10 | 158 |
| C-C motif chemokine 28 (CCL28) | 7 | 14 | 15 | 20 | 630 |
| CD40L receptor (CD40) | 5 | 21 | 15 | 20 | 630 |
| CUB domain-containing protein 1 (CDCP1) | 6 | 24 | 7.5 | 10 | 315 |
| C-X-C motif chemokine 1 (CXCL1) | 6 | 15 | 15 | 10 | 79 |
| C-X-C motif chemokine 5 (CXCL5) | 7 | 13 | 15 | 10 | 315 |
| C-X-C motif chemokine 6 (CXCL6) | 8 | 14 | 15 | 20 | 315 |
| C-X-C motif chemokine 9 (CXCL9 ) | 6 | 12 | 15 | 20 | 315 |
| C-X-C motif chemokine 10 (CXCL10) | 7 | 11 | 15 | 20 | 315 |
| C-X-C motif chemokine 11 (CXCL11) | 7 | 14 | 15 | 20 | 158 |
| Cystatin D (CST5) | 5 | 21 | 15 | 20 | 315 |
| Delta and Notch-like epidermal growth factor-related receptor (DNER) | 5 | 26 | 15 | 20 | 630 |
| Eotaxin-1 (CCL11) | 5 | 14 | 15 | 20 | 630 |
| Eukaryotic translation initiation factor 4E-binding protein 1 (4E-BP1) | 6 | 23 | 15 | 10 | 158 |
| Fibroblast growth factor 5 (FGF-5) | 7 | 14 | 15 | 20 | 630 |
| Fibroblast growth factor 19 (FGF-19) | 6 | 19 | 15 | 20 | 315 |
| Fibroblast growth factor 21 (FGF-21) | 8 | 21 | 3.8 | 5 | 158 |
| Fibroblast growth factor 23 (FGF-23) | 9 | 26 | 7.5 | 10 | 315 |
| Fms-related tyrosine kinase 3 ligand (Flt3L) | 6 | 15 | 15 | 10 | 315 |
| Fractalkine (CX3CL1 ) | 7 | 24 | 15 | 20 | 630 |
| Glial cell line-derived neurotrophic factor (hGDNF) | 7 | 12 | 15 | 20 | 630 |
| Hepatocyte growth factor (HGF) | 6 | 16 | 7.5 | 10 | 315 |
| Interferon gamma (IFN-gamma) | 7 | 17 | 15 | 20 | 630 |
| Interleukin-1 alpha (IL-1 alpha) | 7 | 18 | 15 | 20 | 630 |
| Interleukin-2 (IL-2) | 9 | 16 | 15 | 20 | 630 |
| Interleukin-2 receptor subunit beta (IL-2RB) | 7 | 19 | 15 | 20 | 630 |

(Continued)

| Protein | Repeatability | | Endogenous interference^a^ | | |
| --- | --- | --- | --- | --- | --- |
|  | Intra-assay variation (%) | Inter-assay variation (%) | Hemolysate (g/L) | Lipids (mg/mL) | Bilirubin (µg/mL) |
| Interleukin-4 (IL-4) | 7 | 16 | 15 | 20 | 630 |
| Interleukin-5 (IL-5) | 7 | 17 | 15 | 20 | 630 |
| Interleukin-6 (IL-6) | 6 | 8 | 15 | 20 | 315 |
| Interleukin-7 (IL-7) | 6 | 18 | 15 | 20 | 315 |
| Interleukin-8 (IL-8) | 6 | 15 | 15 | 20 | 79 |
| Interleukin-10 (IL-10) | 7 | 12 | 15 | 20 | 630 |
| Interleukin-10 receptor subunit alpha (IL-10RA) | 6 | 19 | 15 | 20 | 630 |
| Interleukin-10 receptor subunit beta (IL-10RB) | 5 | 31 | 7.5 | 20 | 630 |
| Interleukin-12 subunit beta (IL-12B) | 6 | 16 | 15 | 20 | 630 |
| Interleukin-13 (IL-13) | 14 | 26 | 15 | 20 | 630 |
| Interleukin-15 receptor subunit alpha (IL-15RA) | 6 | 20 | 15 | 20 | 630 |
| Interleukin-17A (IL-17A) | 8 | 17 | 15 | 20 | 630 |
| Interleukin-17C (IL-17C) | 8 | 18 | 15 | 20 | 630 |
| Interleukin-18 (IL-18) | 6 | 19 | 15 | 10 | 315 |
| Interleukin-18 receptor 1 (IL-18R1) | 5 | 26 | 15 | 20 | 630 |
| Interleukin-20 (IL-20) | 7 | 22 | 15 | 20 | 630 |
| Interleukin-20 receptor subunit alpha (IL-20RA) | 6 | 22 | 15 | 20 | 630 |
| Interleukin-22 receptor subunit alpha-1 (IL-22 RA1) | 7 | 23 | 15 | 20 | 630 |
| Interleukin-24 (IL-24) | 6 | 29 | 15 | 20 | 630 |
| Interleukin-33 (IL-33) | 9 | 26 | 15 | 20 | 630 |
| Latency-associated peptide transforming growth factor beta 1 (LAP TGF-beta-1) | 7 | 24 | 7.5 | 20 | 315 |
| Leukemia inhibitory factor (LIF) | 7 | 18 | 15 | 20 | 630 |
| Leukemia inhibitory factor receptor (LIF-R) | 7 | 26 | 15 | 20 | 630 |
| Macrophage colony-stimulating factor 1 (CSF-1) | 5 | 25 | 15 | 20 | 630 |
| Macrophage inﬂammatory protein 1-alpha (MIP-1alpha ) | 6 | 14 | 15 | 20 | 315 |
| Matrix metalloproteinase-1 (MMP-1) | 5 | 19 | 15 | 20 | 630 |
| Matrix metalloproteinase-10 (MMP-10) | 5 | 28 | 15 | 20 | 315 |
| Monocyte chemotactic protein 1 (MCP-1) | 6 | 13 | 15 | 20 | 315 |
| Monocyte chemotactic protein 2 (MCP-2) | 6 | 8 | 15 | 20 | 315 |
| Monocyte chemotactic protein 3 (MCP-3) | 7 | 17 | 15 | 20 | 315 |
| Monocyte chemotactic protein 4 (MCP-4) | 6 | 11 | 15 | 20 | 315 |
| Natural killer cell receptor 2B4 (CD244) | 5 | 24 | 15 | 20 | 630 |
| Neurotrophin-3 (NT-3) | 6 | 13 | 15 | 20 | 630 |
| Neurturin (NRTN) | 9 | 15 | 15 | 20 | 630 |
| Oncostatin-M (OSM) | 5 | 12 | 15 | 20 | 79 |

(Continued)

| Protein | Repeatability | | Endogenous interference^a^ | | |
| --- | --- | --- | --- | --- | --- |
|  | Intra-assay variation (%) | Inter-assay variation (%) | Hemolysate (g/L) | Lipids (mg/mL) | Bilirubin (µg/mL) |
| Osteoprotegerin (OPG) | 6 | 12 | 15 | 20 | 315 |
| Programmed cell death 1 ligand 1 (PD-L1) | 9 | 25 | 15 | 20 | 630 |
| Protein S100-A12 (EN-RAGE ) | 8 | 17 | 15 | 20 | 630 |
| Signaling lymphocytic activation molecule (SLAMF1) | 9 | 21 | 15 | 20 | 630 |
| SIR2-like protein 2 (SIRT2) | 8 | 22 | 15 | 20 | 630 |
| STAM-binding protein (STAMPB) | 5 | 27 | 15 | 20 | 630 |
| Stem cell factor (SCF) | 5 | 20 | 15 | 20 | 630 |
| Sulfotransferase 1A1 (ST1A1) | 6 | 25 | 1.88 | 20 | 630 |
| T-cell surface glycoprotein CD5 (CD5) | 5 | 22 | 15 | 20 | 630 |
| T cell surface glycoprotein CD6 isoform (CD6) | 6 | 23 | 15 | 20 | 630 |
| Thymic stromal lymphopoietin (TSLP) | 6 | 20 | 15 | 20 | 630 |
| TNF-beta (TNFB) | 6 | 22 | 15 | 20 | 630 |
| TNF-related activation-induced cytokine (TRANCE) | 7 | 24 | 15 | 20 | 630 |
| TNF-related apoptosis-inducing ligand (TRAIL) | 5 | 17 | 15 | 20 | 630 |
| Transforming growth factor alpha (TGF-alpha) | 6 | 27 | 15 | 20 | 158 |
| Tumor necrosis factor (Ligand) superfamily member 12 (TWEAK) | 6 | 11 | 15 | 20 | 315 |
| Tumor necrosis factor (TNF) | 9 | 11 | 15 | 20 | 630 |
| Tumor necrosis factor ligand superfamily member 14 (TNFSF14) | 6 | 15 | 15 | 20 | 315 |
| Tumor necrosis factor receptor superfamily member 9 (TNFRSF9) | 5 | 21 | 15 | 20 | 315 |
| Urokinase-type plasminogen activator (uPA) | 5 | 11 | 7.5 | 20 | 315 |
| Vascular endothelial growth factor A (VEGF-A) | 6 | 8 | 15 | 20 | 630 |

^a^ Endogenous interference is assessed by listing highest concentration of each interfering substance without impact on assay performance.

Table S2. The brief description of proteins with more than 65% values below Limit of Detection.

| Number | Proteins | Full names | Types | Function |
| --- | --- | --- | --- | --- |
| 1 | ARTN | Artemin | Growth factor | A neurotrophic factor in the glial cell line-derived neurotrophic factor family of ligands which are a group of ligands within the TGF-beta superfamily of signaling molecules. |
| 2 | IFN-gamma | Interferon-gamma | Cytokine | Innate and adaptive immunity against viral, bacterial and protozoal infections by activating macrophages and inducing Class II major histocompatibility complex (MHC) molecule expression. |
| 3 | IL-10RA | Interleukin 10 Receptor Subunit Alpha | Cytokine | Mediating the immunosuppressive signal of interleukin 10, and thus inhibiting the synthesis of pro-inflammatory cytokines. |
| 4 | IL-13 | Interleukin 13 | Cytokine | Switching to IgG4 and IgE, upregulation of CD23, MHC-II on B cells, induction of CD11b, CD11c, CD18, CD29; CD23, and MHC-II on monocytes, activation of eosinophils and mast cells, recruitment and survival of eosinophils, defense against parasite infections |
| 5 | IL-17A | Interleukin 17A | Cytokine | Induction of pro-inflammatory cytokines, chemokines, and metalloproteases; recruitment of neutrophils |
| 6 | IL-17C | Interleukin 17C | Cytokine | Induction of pro-inflammatory cytokines, chemokines, and metalloproteases |
| 7 | IL-1-alpha | Interleukin 1 alpha | Cytokine | Induction of pro-inflammatory proteins, hematopoiesis, differentiation of TH17 cells |
| 8 | IL-2 | Interleukin 2 | Cytokine | Proliferation of effector T and B cells, development of Treg cells, differentiation and proliferation of NK cells and growth factor for B cells |
| 9 | IL-20 | Interleukin 20 | Cytokine | Role in skin biology |
| 10 | IL-20RA | Interleukin 20 Receptor Subunit Alpha | Cytokine | Involved in several inflammation-related diseases such as psoriasis and colonic inflammation |
| 11 | IL-22.RA1 | Interleukin 22 Receptor Subunit Alpha | Cytokine | Component of one of the receptor for IL20 and IL24 formed by IL22RA1 and IL20RB also signaling through STATs activation. Mediating IL24 antiangiogenic activity as well as IL24 inhibitory effect on endothelial cell tube formation and differentiation. |

| Number | Proteins | Full names | Types | Function |
| --- | --- | --- | --- | --- |
| 12 | IL-24 | Interleukin 24 | Cytokine | Tumor suppression |
| 13 | IL-2RB | Interleukin 2 Receptor Subunit beta | Cytokine | T cell-mediated immune responses |
| 14 | IL-33 | Interleukin 33 | Cytokine | Inducing helper T cells, mast cells, eosinophils and basophils to produce type 2 cytokines. |
| 15 | IL-4 | Interleukin 4 | Cytokine | Induction of TH2 differentiation, IgE class switch, upregulation of class II MHC expression on B cells, upregulation of CD23 and IL-4R, survival factor for B and T cells, role in tissue adhesion and inflammation |
| 16 | IL-5 | Interleukin 5 | Cytokine | Differentiation and function of myeloid cells, increment of chemotactic activity and adhesion capacity on eosinophils, remodeling and wound healing |
| 17 | LIF | Leukemia Inhibitory Factor | Cytokine | Inducing the terminal differentiation of myeloid leukemic cells, thus preventing their continued growth; promoting the growth and cell differentiation of different types of target cells, influencing on bone metabolism, cachexia, neural development, embryogenesis and inflammation. |
| 18 | NRTN | Neurturin | Growth factor | Regulating the survival, development and growth of neurons |
| 19 | TNF | Tumor necrosis factor | Cytokine | Regulation of immune cells and inducing fever, apoptotic cell death, cachexia, inflammation and inhibiting tumorigenesis and viral replication and responding to sepsis via IL-1 and IL-6 producing cells. |
| 20 | TSLP | Thymic stromal lymphopoietin | Cytokine | A critical role in maturation of T cell populations through activation of antigen presenting cells. |

Table S3. Age-specific odds ratios of advanced colorectal neoplasia and non-advanced adenoma for plasma levels of FGF-21 in the replication set

|  | Tertile 1 | Tertile 2 | Tertile 3 | P_trend_^b^ | P_interaction_ |
| --- | --- | --- | --- | --- | --- |
| **Advanced colorectal neoplasia** | |  |  |  |  |
| **Age<65** |  |  |  |  |  |
| No.(Case/Control) | 16/57 | 33/54 | 34/51 | 0.049 |  |
| OR (95%CI)^a^ | Ref. | 2.08 (0.99-4.49) | 2.18 (1.04-4.68) |  |  |
| **Age≥65** |  |  |  |  |  |
| No.(Case/Control) | 6/23 | 12/30 | 24/32 | 0.055 | 0.630 |
| OR (95%CI)^a^ | Ref. | 2.00 (0.57-7.85) | 3.38 (1.02-13.04) |  |  |
|  |  |  |  |  |  |
| **Non-advanced adenoma** | |  |  |  |  |
| **Age<65** |  |  |  |  |  |
| No.(Case/Control) | 8/57 | 21/54 | 18/51 | 0.024 |  |
| OR (95%CI)^a^ | Ref. | 3.19 (1.22-8.97) | 3.30 (1.24-9.43) |  |  |
| **Age≥65** |  |  |  |  |  |
| No.(Case/Control) | 4/26 | 7/30 | 14/32 | 0.071 | 0.837 |
| OR (95%CI)^a^ | Ref. | 2.44 (0.52-13.99) | 3.59 (0.96-16.90) |  |  |

^a^ The model was adjusted for age (continuous variable), sex, BMI (continuous variable),red meat intake, smoking status, alcohol intake, CRC family history of first-degree relative, history of colonoscopy.

^b^ The test of linear trend was performed using a continuous variable with values of 1, 2, 3 for the tertiles in the logistic regression model.

Table S4. Odds ratios of advanced colorectal neoplasia and non-advanced adenoma for plasma levels of FGF-21 with additional adjustment for regular use of NSAID in the replication set

|  | Tertile1 | Tertile2 | Tertile3 | P_trend_^b^ |
| --- | --- | --- | --- | --- |
| **Advanced colorectal neoplasia** | |  |  |  |
| No.(Case/Control) | 22/83 | 45/84 | 58/83 |  |
| OR (95%CI)^a^ | Ref. | 1.89 (1.03-3.56) | 2.14 (1.17-3.99) | 0.019 |
|  |  |  |  |  |
| **Non-advanced adenoma** | |  |  |  |
| No.(Case/Control) | 12/83 | 28/84 | 32/83 |  |
| OR (95%CI)^a^ | Ref. | 2.38 (1.11-5.37) | 2.95 (1.39-6.59) | 0.008 |

^a^ The model was adjusted for age (continuous variable),sex, BMI (continuous variable),red meat intake, smoking status, alcohol intake, CRC family history of first-degree relative, history of colonoscopy and regular use of NSAID.

^b^ The test of linear trend was performed using a continuous variable with values of 1, 2, 3 for the tertiles in the logistic regression model.

Table S5. Diagnostic performance of FGF-21 to detect colorectal cancer and its precursors in the replication set

| Population | AUC (95%CI) | Sensitivity (%) (95%CI) at | |
| --- | --- | --- | --- |
|  |  | 80% specificity | 90% specificity |
| Advanced colorectal neoplasia | 0.61 (0.55-0.67) | 24.8 (17.6-34.4) | 18.4 (12.0-25.6) |
| Colorectal cancer | 0.71 (0.61-0.81) | 45.7 (28.6-65.7) | 37.1 (22.9-54.3) |
| Advanced precancerous lesions | 0.57 (0.50-0.63) | 16.7 (8.9-25.6) | 11.1 (4.4-17.8) |
| Non-advanced adenoma | 0.60 (0.53-0.67) | 20.8 (11.1-41.7) | 12.5 (4.2-22.2) |

Figure S1. The relationship between colorectal carcinogenesis, inflammation and metabolism


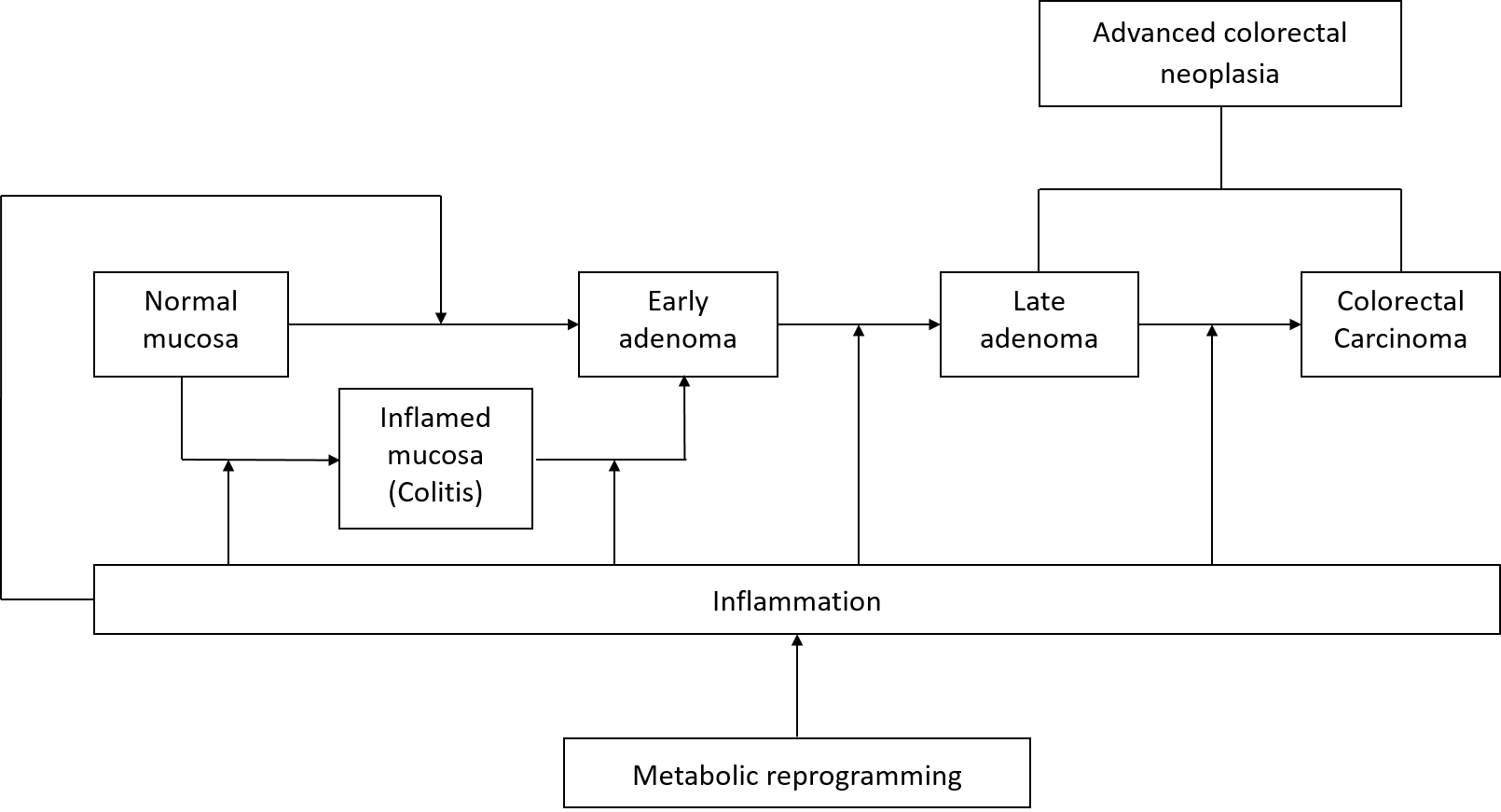


Figure S2. Plasma NPX levels of 8 proteins associated with risk of advanced colorectal neoplasia among advanced neoplasia, non-advanced adenoma and control subjects (*, P<0.05; **, P<0.01; ***, P<0.001; NS, not significant; the NPX levels in control group is reference). Circulating biomarker levels (NPXs) were compared between carriers of advanced colorectal neoplasia and controls as well as carriers of non-advanced adenoma and controls using Wilcoxon Rank Sum Test.

MCP-3, monocyte chemotactic protein 3, CDCP1, CUB domain-containing protein 1, LAP, latency-associated peptide,TGFbeta-1,Transforming Growth Factor Beta 1, IL-6, interleukin-6, TRAIL, TNF-related apoptosis-inducing ligand, CCL4, Chemokine C-C motif ligand 4, SCF, Stem Cell Factor, FGF-21, fibroblast growth factor 21, ACN, advanced colorectal neoplasia, Non-AA, non-advanced adenoma, NS, not significant.


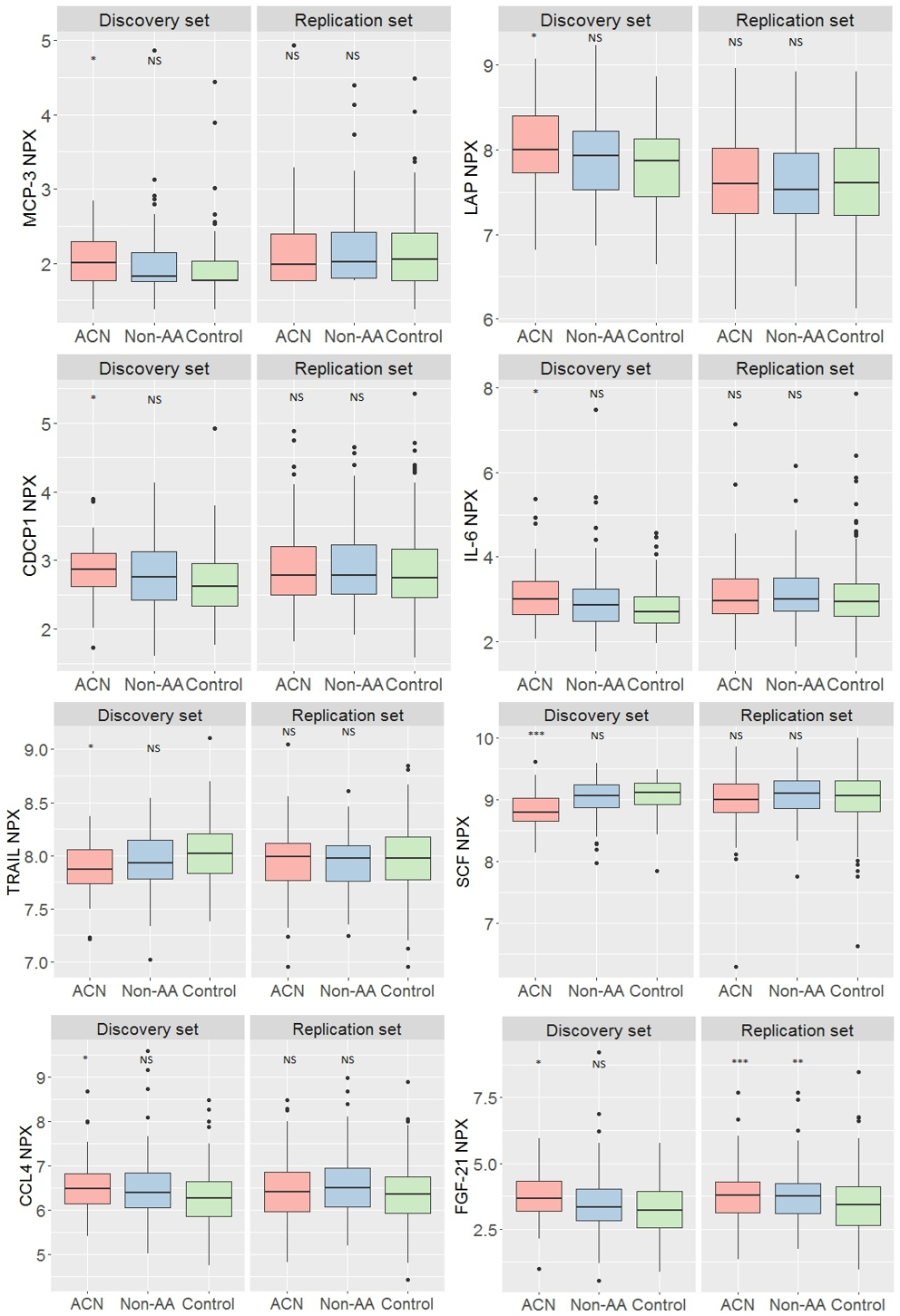

Supplement: Supplementary file 1 — supplementary information_final [file 41416_2018_280_MOESM1_ESM.docx]
